# Impact of Institutional Practices and Surgical Complexity on Sarcoma Surgery Costs: Driving Efficiency in Value-Based Healthcare

**DOI:** 10.3390/cancers16122209

**Published:** 2024-06-13

**Authors:** Georg Schelling, Philip Heesen, Boris Tautermann, Markus Wepf, Barbara Di Federico, Annika Frei, Kim van Oudenaarde, Pietro Giovanoli, Beata Bode-Lesniewska, Gabriela Studer, Bruno Fuchs, on behalf of the Swiss Sarcoma Network

**Affiliations:** 1 Faculty of Health Sciences and Medicine, University of Lucerne, Frohburgstrasse 3, 6002 Luzern, Switzerland; 2 Sarcoma Center & Department of Orthopedics & Trauma, LUKS University Hospital, 6000 Lucerne, Switzerland; 3 Department of Plastic and Reconstructive Surgery, Faculty of Medicine, University of Zurich, 8032 Zurich, Switzerland; 4 Klinik Hirslanden, St. Anna, 6000 Luzern, Switzerland; 5 Sarkomzentrum KSW & Klinik für Orthopädie und Traumatologie, Kantonsspital Winterthur, 8400 Winterthur, Switzerland; 6 Pathologie Enge, Pathology Institute, 8031 Zurich, Switzerland

**Keywords:** sarcoma surgery, healthcare economics, healthcare cost management, institutional resource utilization, surgical complexity, value-based healthcare, quality indicators, tumor complexity scores, predictive cost modelling, benchmarking

## Abstract

**Simple Summary:**

Sarcomas are rare cancers that are complex to treat and can be expensive to manage, especially when considering surgery costs. This research investigates how much sarcoma surgeries cost across three different hospitals in Switzerland and examines what factors might cause these costs to vary. By consistently using the same surgeon for all procedures, the study ensures that differences in surgical expertise do not affect the results, allowing a clear focus on hospital practices and the complexity of tumor impact costs. The findings aim to help hospitals better plan and manage resources, potentially leading to more cost-effective treatments. This study could influence how healthcare systems approach the financial aspects of treating complex cancers, encouraging a shift towards more standardized and economically sustainable practices.

**Abstract:**

Background: Sarcomas present a unique challenge within healthcare systems due to their rarity and complex treatment requirements. This study explores the economic impact of sarcoma surgeries across three Swiss tertiary healthcare institutions, utilizing a consistent surgical approach by a single surgeon to eliminate variability in surgical expertise as a confounding factor. Methods: By analyzing data from 356 surgeries recorded in a real-world-time data warehouse, this study assesses surgical and hospital costs relative to institutional characteristics and surgical complexity. Results: Our findings reveal significant cost variations driven more by institutional resource management and pricing strategies than by surgical techniques. Surgical and total hospitalization costs were analyzed in relation to tumor dignity and complexity scores, showing that higher complexity and malignancy significantly increase costs. Interestingly, it was found that surgical costs accounted for only one-third of the total hospitalization costs, highlighting the substantial impact of non-surgical factors on the overall cost of care. Conclusions: The study underscores the need for standardized cost assessment practices and highlights the potential of predictive models in enhancing resource allocation and surgical planning. By advocating for value-based healthcare models and standardized treatment guidelines, this research contributes to more equitable and sustainable healthcare delivery for sarcoma patients. These insights affirm the necessity of including a full spectrum of care costs in value-based models to truly optimize healthcare delivery. These insights prompt a reevaluation of current policies and encourage further research across diverse geographical settings to refine cost management strategies in sarcoma treatment.

## 1. Introduction

Sarcomas, due to their rarity and the complexity of their treatment, present challenges within healthcare systems [1,2,3]. While advancements in oncological therapies have been made, understanding the economic impact of sarcoma treatments across different hospital settings remains limited [4,5,6,7,8,9]. This gap hinders the effective management and optimization of healthcare resources. Our study addresses this gap by elucidating the variations in surgical and hospital costs of sarcoma treatments within tertiary healthcare environments, supporting the shift towards value-based healthcare models.

Despite clinical advancements, the economic aspects of these treatments, particularly cost variability across different healthcare settings, are underexplored [5,6,10]. Most studies focus on clinical efficacy, often neglecting the substantial economic burdens these treatments impose [11,12]. Our study fills this gap by providing a detailed comparison of surgical and hospital costs and analyzing how institutional characteristics and surgical complexities impact healthcare economics.

Utilizing the Swiss Sarcoma Network (SSN) and the Sarconnector^®^ data warehouse, this study assesses the impact of surgical complexity and institutional characteristics on treatment costs [13]. Leveraging advanced real-world-time data analytics, this research offers insights for more effective resource allocation and enhanced cost-efficiency in managing complex sarcoma surgeries.

The primary objective of this study is to analyze cost disparities across three distinct hospital settings, examining how institutional resources and surgical complexity influence economic outcomes. Importantly, all surgeries were performed by the same surgeon, eliminating variability in surgical expertise and isolating the institutional effects on costs. This study also explores the durations of hospital stays and surgeries to further understand factors contributing to healthcare efficiency and cost management.

## 2. Materials and Methods

This study utilized data from the Swiss Sarcoma Network (SSN), housed in the Adjumed.ch database, and transitioned to the Sarconnector^®^ data warehouse (BF & PH, Zurich, Switzerland) [13,14]. We analyzed a cohort of 356 consecutive patients undergoing surgery for soft tissue or bone tumors across three large Swiss tertiary hospitals. The surgeries, all performed by the same surgeon, aimed to compare surgical and hospital costs, with secondary endpoints including durations of hospital stays and surgical procedures.

Tumor type and dignity were assessed per the 2020 WHO Classification.

Surgical complexity was evaluated using the published Soft-Tissue Tumor Complexity Score (STS-SCS) for soft tissue tumors and a preliminary Bone Tumor Complexity Score (BT-SCS) for bone tumors, capturing the intricacy of each case.

Cost data were meticulously standardized to focus on medically related expenses and extracted from detailed billing records to ensure consistency across the data. These include hospitalization and surgery costs, crucial for evaluating the economic aspects of sarcoma treatment across different institutional settings (Table 1).

This methodological approach ensures comprehensive coverage of cost and care complexities, facilitating an in-depth comparative analysis across the three institutions. Detailed demographic and complexity data, along with raw numbers of tumor entities and categories, are systematically presented in the appendices to support further granular analyses.

Categorical variables were described as numbers (percentage), while numerical variables were described as medians (1st quartile, 3rd quartile). Differences between categorical variables were tested using the Chi-square or Fisher’s exact test. Differences between numerical variables were tested using the Kruskal–Wallis test (for comparisons between >2 groups), Student’s t-test, or the Mann–Whitney test. Pearson correlation coefficients were calculated.

Costs, strictly representing direct medical expenses, were analyzed using multivariable linear regression to adjust for complexity score, tumor dignity, institution, and procedural durations, identifying cost drivers and institutional variations. The beta coefficient and corresponding 95% Confidence Interval (CIs) were reported. The analyses differentiated between impacts derived from numerical and categorical complexity scores to provide a more comprehensive understanding of how each type of scoring influences cost distribution and resource utilization. *p* < 0.05 was considered statistically significant. Statistical analyses were conducted using R statistical software (Version 4.3.1.).

## 3. Results

We have included 356 consecutive patients in our analysis. Of those, 132 were treated in institution A, 121 were treated in institution B, and 103 were treated in institution C. Patient characteristics are presented in Table 2. The median age and the gender distribution of patients across all institutions showed no significant variations between institutions. Tumor dignity varied across the institutions (*p* = 0.03), with Institution C treating a higher proportion of malignant cases. The complexity scores, which reflect the surgical challenge, showed significant differences among the institutions (*p* = 0.002), particularly with Institution C handling more complex cases. The Case Mix Index (CMI), indicative of patient case complexity and resource utilization, also differed significantly, with Institution C having a higher median CMI of 1.09 compared to 0.85 in the others (*p* = 0.001).

### 3.1. Duration of Hospital Stay

In our analysis of 356 patients, we observed significant differences in the median duration of hospital stays across three institutions (Table 3). Institution A reported a median stay of 4 days (interquantile range 2.0 to 8.0), Institution B had a shorter median stay of 3 days (interquantile range 2.0 to 7.0), and Institution C experienced a longer median stay of 5 days (interquantile range 3.0 to 11.5), with these variations reaching statistical significance (*p* < 0.001). Additionally, the duration of hospital stays varied significantly with tumor dignity; patients with benign tumors had a median stay of 2 days (interquantile range 2.0 to 3.5), those with intermediate tumors stayed for 4 days (interquantile range 2.0 to 7.0), and patients with malignant tumors stayed for 8 days (interquantile range 5.0 to 13.0). A clear trend of increasing hospital stay duration with higher complexity scores was also observed; the median stay was 2 days for scores 1 and 2, 4 days for score 3, and 9 days for score 4 (*p* < 0.001 for all). These findings underscore the impact of institutional characteristics, tumor dignity, and complexity on the length of hospitalization.

**Figure 1:**
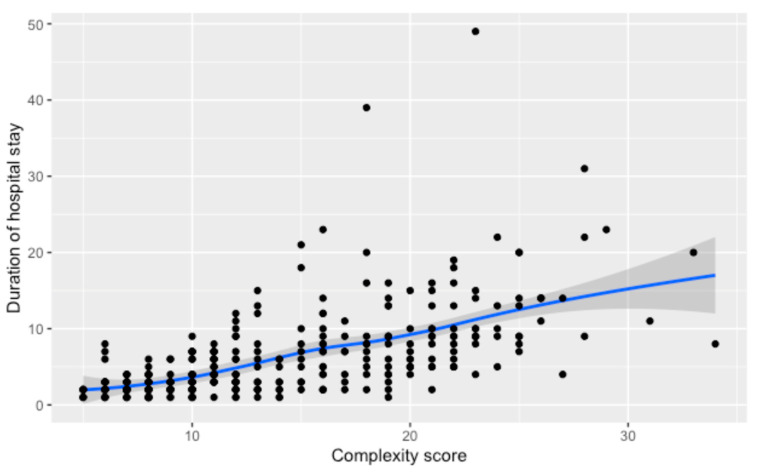
Relationship between the numerical complexity score and duration of hospital stay. Loess smoothing was applied to allow for a potential non-linear relationship.

The relationship between complexity score and hospital stay duration is depicted in Figure 1, showing a substantial, statistically significant correlation coefficient of 0.70 (*p* < 0.001). The trend line demonstrates an increase in stay duration as complexity scores rise, with the confidence interval indicating the estimate’s precision.

### 3.2. Duration of Surgery

The median duration of surgery across the study cohort was 89.5 min, with an interquartile range of 56 to 165 min, and no significant differences were observed between institutions (*p* = 0.76). A detailed analysis by tumor dignity revealed significant variability, with benign tumors requiring the least time at a median of 60 min, intermediate tumors at 86 min, and malignant tumors demanding the most time at a median of 150.5 min (*p* < 0.001). Surgery duration also increased with tumor complexity, rising from a median of 44 min for the least complex cases (score 1) to 163 min for the most complex cases (score 4), substantiating the strong influence of tumor complexity on surgical time (*p* < 0.001) (Table 4).

**Figure 2:**
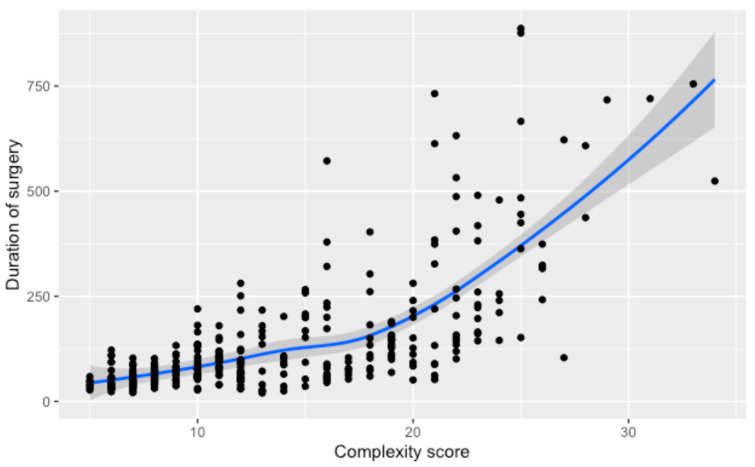
Relationship between the numerical complexity score and the duration of surgery. Loess smoothing was applied to allow for a potential non-linear relationship.

Comparing the numerical complexity score and the duration of surgery, we found a non-linear relationship. Initial increases in complexity correlate with a linear rise in surgical time, but for scores beyond 20, the duration sharply ascends, as demonstrated by the steepening curve. A regression analysis shows that surgeries for patients with a complexity score higher than 20 take, on average, 255 additional minutes compared to those with lower scores (95% CI; 223,287), *p* < 0.001, underscoring the substantial impact of high complexity on surgery duration (Figure 2).

### 3.3. Costs of Surgery

The median cost of tumor surgery within the patient cohort was CHF 3519, with an interquartile range from CHF 2502 to CHF 5945. Institution-specific analysis revealed significant cost differences: CHF 3118 for Institution A, CHF 3408 for Institution B, and CHF 4611 for Institution C, with statistically significant variations among them (*p* = 0.001). When examining costs by tumor dignity, benign surgeries were least expensive at a median of CHF 2570, while malignant surgeries were most costly at CHF 5502, showing significant cost differences (*p* < 0.001). Furthermore, costs rose with increasing complexity scores, from CHF 2207 for the simplest surgeries to CHF 5964 for the most complex, reflecting the substantial impact of complexity on surgical costs (*p* < 0.001) (Table 5).

**Figure 3:**
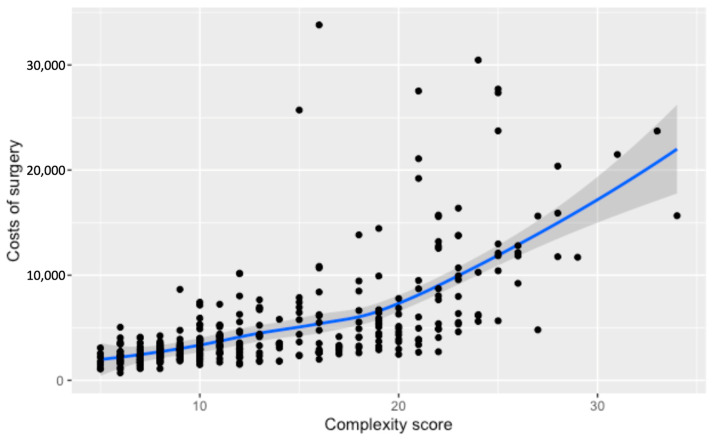
Relationship between the numerical complexity score and the costs of surgery (c). Loess smoothing was applied to allow for a potential non-linear relationship.

A non-linear rise in surgery costs with increasing complexity scores is depicted in Figure 3, showcasing a moderate climb until reaching a score of 20. Beyond this point, there’s a marked and notable upsurge in expenses. Cases that exceed a complexity score of 20 exhibit an average cost increase of CHF 7627, signaling a substantial elevation in the financial burden for surgeries of greater complexity.

**Figure 4:**
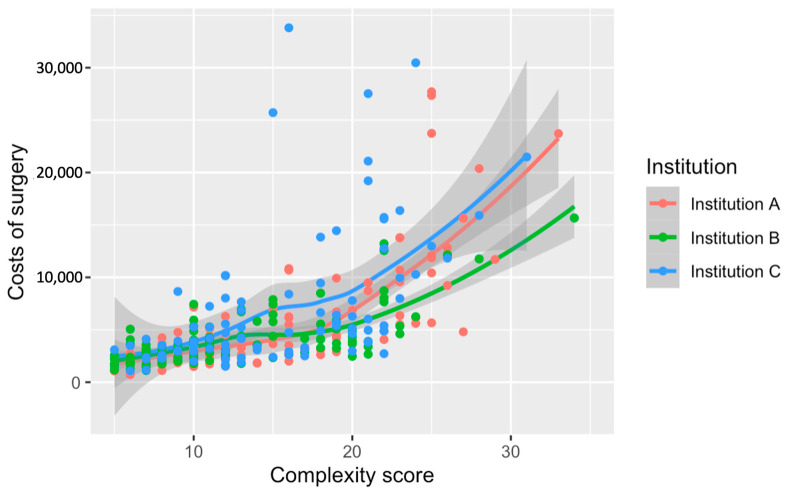
Relationship between the numerical complexity score and costs of surgery stratified by institution. Loess smoothing was applied to allow for a potential non-linear relationship.

The comparison across institutions depicts a divergence in surgery costs in relation to complexity scores (Figure 4). Institution C’s curve is positioned above the rest, indicating consistently higher costs throughout the complexity spectrum. The separation between the institutional curves becomes pronounced at higher complexity levels, particularly after surpassing a score of 20. These cost discrepancies among institutions have been statistically substantiated, with Institution C incurring significantly higher expenses than A and B (*p* < 0.001). The visualization underscores the pronounced impact that both the complexity of surgical procedures and institutional factors have on the cost of care.

### 3.4. Total Costs for Hospitalization

The median total cost for all patients involved in the study was CHF 10,378, with an interquartile range from CHF 6865 to CHF 18,806. Institutionally, median total costs varied, with Institution A at CHF 8827, Institution B at CHF 10,564, and Institution Cat at CHF 11,990, indicating a marginal statistical difference (*p* = 0.05). Total costs demonstrated a significant disparity with tumor dignity, with benign tumors costing CHF 6828, intermediate CHF 10,062, and malignant CHF 18,374 (*p* < 0.001). A statistically significant gradient was also seen with complexity scores, showing CHF 5650 for score 1, escalating to CHF 22,779 for score 4 (*p* < 0.001). Adjusted for variables such as complexity score, tumor dignity, and durations of surgery and hospital stay, Institution B’s total costs were on average CHF 1737 higher than A, and costs in Institution C were CHF 1426 lower compared to B (Table 6).

**Figure 5:**
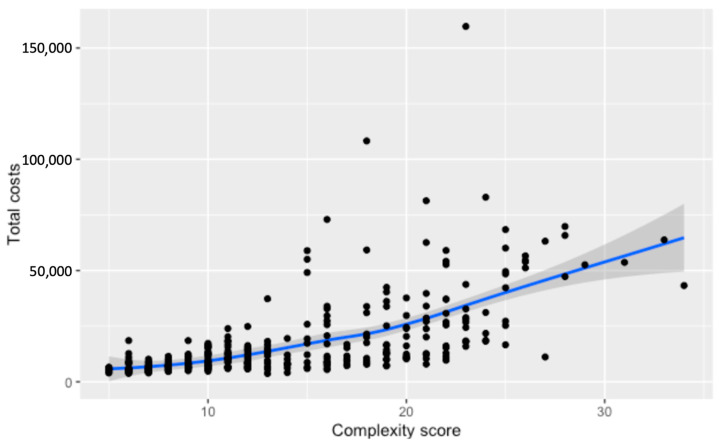
Relationship between the numerical complexity score and total costs for hospitalization. Loess smoothing was applied to allow for a potential non-linear relationship.

**Figure 6:**
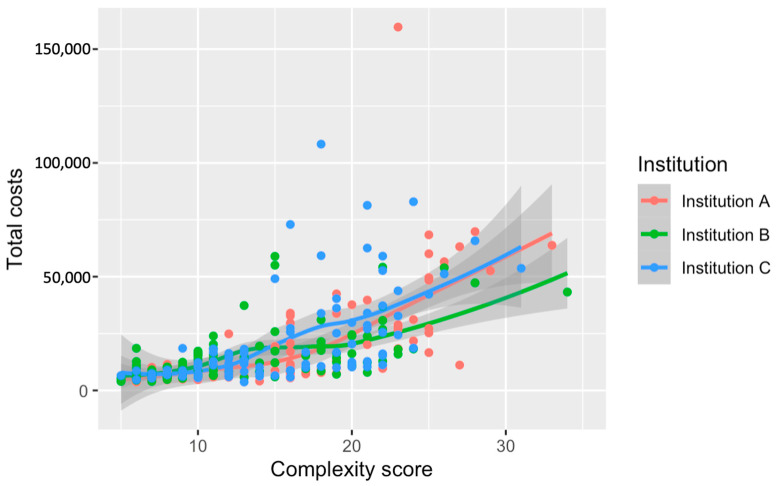
Relationship between the numerical complexity score and total costs for hospitalization stratified by institution. Loess smoothing was applied to allow for a potential non-linear relationship.

Total costs demonstrate a progressive increase in association with higher complexity scores. A steady incline in costs becomes more pronounced at higher complexity levels, reflecting the growing financial impact of more intricate surgical cases. When Loess smoothing is applied, the data reveal a generally upward, albeit less pronounced, trend in total costs with rising complexity, contrasting with the more marked effects on surgery duration and direct surgery costs (Figure 5 and Figure 6).

## 4. Discussion

Our investigation revealed significant variations in financial and operational metrics across three healthcare institutions in one country, particularly emphasizing the role of surgical complexity and tumor characteristics in influencing cost metrics. All surgeries were performed by the same surgeon, thus eliminating variations in surgical expertise and technique as potential confounders. While this approach helps isolate institutional factors such as resource utilization, efficiency, and possible institutional policies on pricing strategies, it is also crucial to recognize that variations in surgical expertise among different surgeons can significantly impact economic policies and overall healthcare costs. Future research should consider the influence of surgeon expertise on cost variability to provide a more comprehensive understanding of economic factors in healthcare.

The median total costs for all patients were CHF 10,378, with Institution C exhibiting notably higher expenses at CHF 11,990, suggesting variations in institutional resource allocation and management efficiency. While surgical costs averaged CHF 3519, they constituted only about one-third of the total hospitalization costs, underscoring the significant role of postoperative care and extended hospital stays. Furthermore, our analysis demonstrated substantial disparities in costs based on tumor dignity, with malignant tumors resulting in the highest expenditures at a median of CHF 18,374, directly correlating tumor severity and treatment complexity with increased healthcare expenditures. Complexity scores strongly correlated with both surgical and hospitalization costs, showing a non-linear increase in expenses as complexity scores exceeded 20, emphasizing the critical need for precise surgical and resource planning.

Our study’s insights into the economic impact of institutional policies and resource management on sarcoma surgery costs align with and expand upon existing literature. Kuek et al. discuss how surgical costs influence the quality of care across economic environments, underscoring the role of cost in treatment accessibility and quality [5]. Similarly, Morattel et al. illustrate how unplanned surgeries escalate costs, supporting our emphasis on the economic benefits of strategic preoperative planning [15]. Alamanda et al. quantify the financial impact of reexcising incompletely excised sarcomas, highlighting the economic advantages of precise surgical interventions [16]. These studies collectively advocate for standardized practices to mitigate cost disparities, resonating with our observations of non-linear cost increases with higher complexity scores. Additionally, our research underscores the potential of predictive modeling and value-based healthcare frameworks to optimize resource allocation [10].

Our examination of cost variations across three institutions revealed significant disparities that are more reflective of institutional policies and system-level efficiencies than differences in surgical expertise. Differences in the use of surgical supplies, the adoption of new technologies, and the extent of ancillary services provided might account for the observed variations in expenditures. While our approach helps isolate these factors, it is important to note that variations in surgical expertise among different surgeons can significantly impact economic policies and overall healthcare costs. Future research should consider the influence of surgeon expertise on cost variability to provide a more comprehensive understanding of economic factors in healthcare.

The management of high-complexity cases provides further insights into how institutional protocols and the availability of specialized support services influence costs. Despite uniform surgical expertise, variation in expenses could be attributed to differing approaches in preoperative care, postoperative management, and the use of intensive care facilities. This underscores the necessity for standardized guidelines that can streamline care pathways across institutions, potentially reducing unnecessary variations in patient care and associated costs.

Moreover, this study reinforces the importance of establishing clear and consistent definitions of what constitutes surgical and hospital costs across different settings. A standardized approach to cost categorization and accounting would enhance the ability to compare and benchmark costs meaningfully across institutions. This is particularly relevant in settings where surgical interventions are performed by the same healthcare professionals, highlighting that differences in costs are largely driven by institutional practices and policies rather than individual surgeon performance.

The disparities in surgical costs across institutional settings and varying complexity scores highlight the necessity for refined surgical planning and patient care, underscoring the urgent need for targeted resource allocation strategies. Given the unique and diverse nature of sarcoma surgeries, our study advocates for harmonized accounting practices that accommodate the exceptional nature of each surgical case, promoting cost management that reflects the true complexity and needs of each procedure.

Our findings strongly support the advancement of value-based healthcare initiatives, suggesting that integrating predictive models based on complexity scores could significantly enhance preoperative planning and patient outcomes. Such models could serve as a cornerstone for developing more streamlined care pathways, particularly for managing complex and severe cases. Moreover, the clear trends in cost relative to complexity and tumor severity provide a robust framework for predictive budgeting and resource planning.

Furthermore, the potential for policy adjustments based on our findings is substantial. Policymakers are encouraged to consider the benefits of shifting towards a value-based healthcare model that prioritizes efficiency and cost-effectiveness, especially in the treatment of high-complexity surgical cases [17,18,19,20,21]. By adopting policies that foster the implementation of such models, healthcare systems can better manage expenditures while improving clinical outcomes.

Our study, while offering insightful contributions to understanding surgical cost variations in sarcoma treatment, has inherent limitations that must be acknowledged. One significant challenge is the lack of uniformity in cost assessment across the different institutions. The definitions of surgical and hospital costs varied, which might have influenced the comparability of our findings. Although efforts were made to standardize these definitions, the subtleties in cost allocation and accounting practices could lead to different interpretations, potentially affecting the study’s outcomes. Future research should aim to refine these definitions and develop a more standardized approach to cost assessment in healthcare settings to enhance comparability [22].

Additionally, the geographical and cultural homogeneity of the institutions involved—all located within a small country with minimal cultural and demographic differences—may limit the extrapolation of our results to the international community. While our study is based on data from Swiss hospitals, the findings highlight important factors that could be relevant to other healthcare systems with the necessary adaptations. The similar patient profiles across these institutions might obscure broader variations that could become apparent in a more diverse sample. Despite this, the evident cost disparities found within such a homogenous group underscore the intrinsic differences in institutional cost management and highlight the need for further investigation into the underlying causes of these variations.

These limitations underscore the complexity of healthcare cost analysis and point to the necessity for ongoing research that includes a broader range of institutions and more diverse geographical settings. This would help to validate the findings and potentially reveal additional insights that could inform policy changes and lead to more equitable and efficient healthcare delivery.

## 5. Conclusions

This study demonstrates significant cost variations in sarcoma surgery, primarily driven by institutional practices rather than surgical expertise. By using a consistent surgeon across all cases, we isolated institutional influences, such as resource utilization, management efficiency, and pricing strategies, which significantly affect healthcare expenditures. Notably, surgical costs constituted only a third of total hospitalization costs, emphasizing the need for comprehensive cost assessments that encompass the full spectrum of care.

We advocate for the adoption of standardized treatment guidelines and value-based healthcare models that integrate full-spectrum cost considerations, enhancing cost-effectiveness and sustainability in managing complex medical conditions. Further research should account for variations in surgical expertise and include geographical settings to validate and refine these strategies, ensuring equitable and efficient healthcare delivery for conditions like sarcoma.

## Figures and Tables

**Table 1:** Definition of costs.

Category	Subcategory	Description
**Hospitalization Costs**	**Individual Costs**	
	Total Individual Costs	Direct individual costs specifically attributed to the patient’s hospital stay.
	Medication Costs	Costs specifically incurred for medications uniquely used in this case during hospitalization.
	Material and Implant Costs	Costs for materials and implants specifically used for this patient during hospitalization.
	**Common Costs**	
	Total Common Costs	General overhead costs that are shared among various patients but are part of the hospitalization expenses.
	Operating Room costs	Costs associated with the use of operating rooms include infrastructure, personnel, and minor materials shared among surgical specialists.
	Anesthesia and Recovery Room Costs	Costs related to anesthesia services and the recovery room are applicable to multiple patients during hospitalization.
	Intensive Care Costs	Costs for intensive care are shared across patients needing such services during hospitalization.
	Medical (Fraternity) Staff Costs	Costs for surgical and general medical staff involvement spread over multiple cases during hospitalization.
	Nursing Costs	Nursing costs at the ward level, shared among patients during hospitalization.
**Surgical Costs**	**Direct Costs**	
	Operating Room Usage Costs	Costs associated with operating room time (patients arrival until leaving), including all aspects of OR management and nursing costs as well as apportionment of indirect costs.
	Surgical Staff Costs	Direct costs for medical fraternity present in the operating room, calculated based on surgical service time.
	Anesthesia Costs	Anesthesia costs during surgery and for the (anesthesia-led) recovery room. During surgery, the cost is calculated in one of four categories, depending on the type of anesthesia. In the recovery room, a minute-based cost is calculated.
	**Indirect Costs**	
	Anesthesia Costs	Apportionment of indirect costs.

Table 1 summarizes hospitalization and surgery costs from sarcoma surgeries at three Swiss healthcare institutions, detailing: ***Individual costs:*** Costs are directly and uniquely attributable to a specific patient’s treatment. These are the expenses incurred that can be exclusively associated with a single case. ***Common costs:*** Costs incurred by the healthcare facility that are shared among multiple patients. These costs are not exclusive to any single patient’s care and are distributed across various treatments and services. ***Direct (surgical) costs:*** These are costs in a surgical setting that are directly associated with the patient’s surgery, such as operating room usage and staffing. ***Indirect (surgical) costs:*** These costs are not directly tied to the actual performance of the surgery but are necessary to support the surgical process.

**Table 2:** Patient characteristics stratified by institution.

	Overall n = 356	Institution A n = 132	Institution B n = 121	Institution C n = 103	*p*-Value
**Age**	51.0 (34.0, 65.0)	52.5 (37.0, 67.0)	51.0 (34.0, 62.0)	48.0 (33.0, 63.0)	0.33
**Female**	157 (44.1%)	56 (42.4%)	45 (46.3%)	45 (43.7%)	0.82
**Dignity** Benign Intermediate Malignant	115 (32.3%) 80 (22.5%) 161 (45.2%)	42 (31.8%) 31 (23.5%) 59 (44.7%)	50 (41.3%) 26 (21.5%) 45 (37.2%)	23 (22.3%) 23 (22.3%) 57 (55.4%)	0.03
**Complexity score** 1 2 3 4	25 (7.0%) 77 (21.6%) 110 (30.9%) 118 (33.1%)	8 (6.0%) 34 (25.8%) 33 (25.0%) 46 (34.8%)	14 (11.6%) 31 (25.6%) 40 (33.1%) 29 (24.0%)	3 (2.9%) 12 (11.7%) 37 (35.9%) 43 (41.7%)	0.002
**Case mix index (CMI)**	0.86 (0.71, 1.37)	0.85 (0.70, 1.38)	0.85 (0.70, 1.30)	1.09 (0.77, 1.63)	0.001

Numerical variables presented as Median (1st quartile, 3rd quartile).

**Table 3:** Duration of stay, tumor dignity, and complexity score. Results are reported as Median (1st quartile, 3rd quartile).

Characteristic	Overall	Institution A	Institution B	Institution C	*p*-Value
Duration of Stay	4.0 (2.0–8.0)	4.0 (2.0–8.0)	3.0 (2.0–7.0)	5.0 (3.0–11.5)	<0.001
**By Tumor Dignity** -benign -intermediate -malignant	2.0 (2.0–3.5) 4.0 (2.0–7.0) 8.0 (5.0–13.0)	2.0 (2.0, 3.0) 4.0 (3.0, 7.0) 8.0 (4.5, 13.0)	2.0 (1.0, 3.0) 3.0 (2.0, 6.0) 7.0 (5.0, 9.0)	3.0 (2.0, 4.0) 4.0 (2.5, 7.5) 9.0 (5.0, 15.0)	<0.001
**By Complexity Score** -Score 1 -Score 2 -Score 3 -Score 4	2.0 (1.0–3.0) 2.0 (2.0–3.0) 4.0 (2.25–7.0) 9.0 (5.0–13.0)	1.5 (1.0, 2.25) 2.0 (2.0, 3.0) 4.0 (3.0, 7.0) 9.0 (5.25, 13.0)	2.0 (2.0, 3.0) 2.0 (1.0, 2.5) 4.0 (2.0, 7.0) 7.0 (5.0, 9.0)	2.0 (2.0, 2.0) 3.0 (2.0, 4.0) 3.0 (2.0, 6.0) 10.0 (5.5, 14.5)	<0.001

Numerical variables are presented as Median (1st quartile, 3rd quartile).

**Table 4:** Overall duration of surgery, tumor dignity, and complexity score. Results are reported as Median (1st quartile, 3rd quartile).

Characteristics	Overall	Institution A	Institution B	Institution C	*p*-Value
**Duration of surgery**	89.5 (56.0–165.0)	83.5 (61.0, 176.0)	89.0 (56.0, 147.0)	90.0 (53.0, 199.5)	0.76
**Tumor Dignity** -benign -intermediate -malignant	60.0 (43.0–82.3) 86.0 (58.0–123.5) 150.5 (80.3–277.5)	62.5 (40.0–75.8) 82.0 (61.5–120.0) 185.0 (87.0–374.0)	57.5 (47.3, 94.8) 87.5 (58.8, 108.8) 144.0 (93.0, 237.0)	60.0 (41.5, 89.3) 90.0 (40.0, 167.0) 147.0 (60.5, 255.5)	<0.001
**Complexity Score** -Score 1 -Score 2 -Score 3 -Score 4	44.0 (33.0–59.0) 57.5 (43.3–75.8) 90.0 (58.0–136.0) 163.0 (103.8–336.0)	46.5 (38.0–78.8) 62.0 (41.0–75.8) 77.0 (62.0–106.0) 198.5 (112.0–383.5)	48.5 (38.0, 58.8) 55.0 (46.5, 73.0) 106.0 (74.0, 152.5) 144.0 (93.0, 246.0)	32.0 (31.0, 36.0) 52.0 (42.0, 81.0) 77.5 (41.3, 161.8) 158.0 (113.0, 281.0)	<0.001

**Table 5:** Overall costs for surgery, tumor dignity, and complexity score. Results are reported as Median (1st quartile, 3rd quartile).

Factor	Overall [CHF]	Institution A [CHF]	Institution B [CHF]	Institution C [CHF]	*p*-Value
**Costs Surgery**	3519 (2502–5945)	3118 (2228–6142)	3408 (2464–5050)	4611 (2957–7885)	0.001
**Total Cost by Dignity** -benign -intermediate -malignant	2570 (1998–3321) 3545 (2571–5062) 5502.7 (3270–10,281)	2259 (1868–2743) 3317 (2385–4325) 6137 (3076–10694)	2612 (2016–3578) 3262 (2332–4001) 4616 (3709–7871)	3087 (2532–3841) 4693 (3388–7044) 5349 (3074–12,762)	<0.001
**Total Cost by Complexity Score** -Score 1 -Score 2 -Score 3 -Score 4	2207 (1584–2760) 2431 (1969–3035) 3531 (2627–5250) 5964 (3948–11,747)	2247 (1455–2709) 2259 (1889–2915) 3010 (2366–3796) 6300 (4108–11,491)	2041 (1689–2511) 2473 (2064–2987) 3846 (2918–5375) 4616 (3709–8039)	3087 (2074–3278) 2990 (2342–3631) 3767 (2924–5559) 6291 (4811–13,715)	<0.001

**Table 6:** Total costs for hospitalization, tumor dignity, and complexity score.

Factor	Overall Median Cost [CHF]	Institution A [CHF]	Institution B [CHF]	Institution C [CHF]	*p*-Value
**Total Costs**	10,378 (6865–18,806)	8827 (6116–16,719)	10,564 (6852–17,310)	11,990 (8121–26,390)	0.005
**Cost by Tumor Dignity** –Benign –Intermediate –malignant	6828 (5419–8739) 10,062 (7119–13,004) 18,374 (10,160–37,228)	5825 (4939–7291) 8745 (6567–12,226) 20,123 (8878–38,691)	7189 (5569–10,875) 9616 (6954–12,449) 18,079 (12,877–31,054)	7363 (6161–8816) 12,221 (9467–16,404) 18,627 (10,292–40,374)	<0.0001
**Cost by Complexity Score** –Score 1 –Score 2 –Score 3 –Score 4	5650 (5217–8007) 6568 (5431–7814) 10,367 (7118–15,369) 22,779 (11,752–39,198)	5535 (4988–5708) 5942 (4939–7761) 8705 (6619–12,186) 23,526 (10,692–41,764)	5882 (5235–9202) 6852 (5604–7775) 13,817 (8459–17,281) 18,079 (12,368–26,846)	6525 (5609–7568) 7312 (6120–8001) 10,876 (7019–14,072) 25,677 (12,579–43,020)	<0.001

## Data Availability

The data presented in this study are available upon request from the corresponding authors.
